# Supplementary material for: Diverse bacteriophages for biocontrol of ESBL- and AmpC-β-lactamase-producing E. coli
Source: iScience. 2024 Jan 17;27(2):108826. doi: 10.1016/j.isci.2024.108826 (PMC10844046; doi:10.1016/j.isci.2024.108826)

iScience, Volume *27*

Supplemental information

Diverse bacteriophages for biocontrol

of ESBL- and AmpC-β-lactamase-producing *E. coli*

Amira R. Vitt, Anders Nørgaard Sørensen, Martin S. Bojer, Valeria Bortolaia, Martine C. Holst Sørensen, and Lone Brøndsted

Supplementary information

Table S2: The ability of each phage to infect diverse phylogroup and ST, related to Figure 4.
Table S3: List of primers for deletions of common bacteriophage receptor genes in *E. coli* strain ECOR4, related to Figure 5.
Table S4: List of primers for control of the deletion of genes expressing common bacteriophage receptor genes in *E. coli* strain ECOR4, related to Figure 5.
Figure S1: Protein sequence alignment of gp38 corresponding to the chaperone encoded by T4, related to Figure 5.
Figure S2: Protein sequence alignment of the tail fibers corresponding to the adhesins encoded by gp38 homologues of the phages T2 and T6, related to Figure 5.
Figure S3: Protein sequence alignment of gp37 of T4 and its homologues, related to Figure 5.

References

1. Vitt, A.R., Sørensen, M.C.H., Bortolaia, V., and Brøndsted, L. (2023). A Representative Collection of Commensal Extended-Spectrum- and AmpC-β-Lactamase-Producing Escherichia coli of Animal Origin for Phage Sensitivity Studies. PHAGE: Therapy, Applications, and Research *4*, 35–45. 10.1089/phage.2023.0002

Table S2: The ability of each phage to infect diverse phylogroup and STs, related to Figure 4.

Phage	Number of infected strains	Phylo-groups	STs based on MLST analysis
AV101	9	A, B1, C	ST-1800, ST-2040, ST-6206, ST-641, ST-88
AV102	4	B1, C	ST-162, ST-1431, ST-23
AV103	4	B1, C	ST-23, ST-641, ST-6254, ST-453
AV104	2	D	ST-69, ST-4243
AV105	2	D	ST-69, ST-4243
AV106	1	C	ST88
AV108	5	A, B1	ST-295, ST-10, ST-154, ST-6206
AV109	9	B1, C	ST-88, ST-7614, ST-4663, ST-295
AV110	48	A, B1, B2, C, G	ST-10, ST-101, ST-117, ST-1431, ST-154, ST-155, ST-156, ST-23, ST-295, ST-345, ST-3564, ST-429, ST-4580, ST-4663, ST-4980, ST-58, ST-665, ST-718, ST-75, ST-88
AV111	46	A, B1, C, D, E, F	ST-10, ST-1147, ST-1640, ST-23, ST-2607, ST-354, ST-4243, ST-453, ST-4663, ST-4980, ST-57, ST-58, ST-641
AV112	37	A, B1, C, D	ST-10, ST-101, ST-1011, ST-1431, ST-154, ST-155, ST-156, ST-1800, ST-2040, ST-295, ST-3564, ST-4580, ST-48, ST-4980, ST-58, ST-718, ST-75, ST-877, ST-88
AV113	25	A, B1, C, E	ST-295, ST-10, ST-101, ST-1431, ST-154, ST-155, ST-156, ST-1800, ST-295, ST-3564, ST-48, ST-57, ST-58, ST-718, ST-877, ST-88
AV114	29	A, B1, C, G	ST-117, ST-155, ST-165, ST-295, ST-3564, ST-453, ST-4663, ST-4980, ST-718, ST-7614, ST-88
AV115	33	A, B1, C, G	ST-101, ST-117, ST-1431, ST-165, ST-295, ST-3564, ST-453, ST-4663, ST-58, ST-88
AV116	17	A, B1, C	ST-101, ST-1431, ST-154, ST-155, ST-156, ST-1800, ST-2040, ST-295, ST-4580, ST-6206, ST-88
AV117	16	A, B1, C	ST-101, ST-1431, ST-154, ST-155, ST-156, ST-1800, ST-2040, ST-295, ST-4580, ST-6206, ST-88
AV118	9	A, B1, C	ST-155, ST-1850, ST-4580, ST-88
AV119	55	A, B1, B2, C, D, E, F	ST-10, ST-1147, ST-115, ST-131, ST-1640, ST-23, ST-355, ST-367, ST-4243, ST-453, ST-4663, ST-4980, ST-57, ST-6858, ST-69, ST-877, ST-88
AV120	50	A, B1, B2, C, D, E	ST-10, ST-131, ST-1640, ST-23, ST-2607, ST-367, ST-4243, ST-453, ST-4663, ST-4980, ST-57, ST-58, ST-602, ST-877, ST-88
AV121	26	Clade I, A, B1, B2, C, D	ST-115, ST-131, ST-2309, ST-355, ST-4243, ST-453, ST-4980, ST-770, ST-88
AV122	16	Clade I, B1, C, E	ST-101, ST-1431, ST-154, ST-155, ST-1640, ST-4580, ST-770, ST-877, ST-88
AV123	51	A, B1, B2, C, D, E	ST-101, ST-1056, ST-1147, ST-1286, ST-131, ST-1463, ST-154, ST-155, ST-162, ST-1800, ST-1850, ST-2040, ST-23, ST-2607, ST-38, ST-4243, ST-429, ST-4580, ST-4663, ST-4980, ST-57, ST-602, ST-6206, ST-718, ST-88
AV124	2	C	ST-23, ST-88
AV125	84	A, B1, B2, C, D, E	ST-10, ST-101, ST-1011, ST-1056, ST-1147, ST-115, ST-1431, ST-1463, ST-154, ST-155, ST-156, ST-162, ST-1640, ST-1800, ST-1850, ST-2040, ST-23, ST-2309, ST-2607, ST-295, ST-345, ST-3564, ST-367, ST-38, ST-4243, ST-429, ST-4580, ST-4663, ST-4980, ST-57, ST-602, ST-6206, ST-6254, ST-718, ST-88
AV126	65	Clade I, A, B1, B2, C, D, E	ST-770, ST-10, ST-101, ST-1011, ST-115, ST-1463, ST-154, ST-155, ST-1640, ST-1800, ST-1850, ST-2040, ST-23, ST-2607, ST-295, ST-345, ST-351, ST-38, ST-4243, ST-429, ST-4580, ST-4980, ST-57, ST-602, ST-6206, ST-718, ST-877, ST-88
AV127	13	A, B1, C	ST-1463, ST-155, ST-23, ST-4980, ST-4980, ST-57, ST-602, ST-88
AV128	8	A, B1, D, E	ST-101, ST-1640, ST-2197, ST-38, ST-57
AV129	12	A, B1, C, D, E	ST-1640, ST-23, ST-2952, ST-4243, ST-4980

Table S3: List of primers for deletions of common bacteriophage receptor genes in *E. coli* strain ECOR4, related to Figure 5.

**Primers for deletion fragment amplification**	
ompW-Up	ATGAAAAAGTTAACAGTGGCGGCTTTGGCAGTAACAACTCTTCTCTCTGG*AATTAACCCTCACTAAAGGGCG*
ompW-Dw	TTAAAAACGATATCCTGCTGAGAGCATAAACACCCACGGATCGAGGCGTA*TAATACGACTCACTATAGGGCTC*
tsx-Up	ATGAAAAAAACATTACTGGCAGCCGGTGCGGTACTGGCGCTCTCTTCGTC*AATTAACCCTCACTAAAGGGCG*
tsx-Dw	TCAGAAGTTGTAACCTACTACCAGGTAACCACCCCAGCCGGTAGAGCGAA*TAATACGACTCACTATAGGGCTC*
waaC-Up	ATGCGGGTTTTGATCGTTAAAACATCGTCGATGGGCGATGTTCTCCATAC*AATTAACCCTCACTAAAGGGCG*
waaC-Dw	TTAAATCAATGTGATTTTTTTATAAACAGCCGCTGCTTCTAAATTATTTA*TAATACGACTCACTATAGGGCTC*
tonB-Up	ATGACCCTTGATTTACCTCGCCGCTTCCCCTGGCCGACGTTACTTTCGGT*AATTAACCCTCACTAAAGGGCG*
tonB-Dw	TTACTGAATTTCGGTGGTGCCGTTAATTTTAAACAGGATATTCACCACAA*TAATACGACTCACTATAGGGCTC*
waaR-Up	ATGAATGAATTTATAAAAGAACGGTTTTCGTATTTAGCAGATAATAAAAA*AATTAACCCTCACTAAAGGGCG*
waaR-Dw	TTATTTCTTAAGCTTGTACTTAATTAATGAAGTTATGCCTTTTATATACT*TAATACGACTCACTATAGGGCTC*
ompF-Up	ATGATGAAGCGCAATATTCTGGCAGTGATCGTCCCTGCTCTGTTAGTAGC*AATTAACCCTCACTAAAGGGCG*
ompF-Dw	TTAGAACTGGTAAACGATACCCACAGCAACGGTGTCGTCTGAACCTACGC*TAATACGACTCACTATAGGGCTC*
fadL-Up	ATGAGCCAGAAAACCCTGTTTACAAAGTCTGCTCTCGCAGTCGCAGTGGC*AATTAACCCTCACTAAAGGGCG*
fadL-Dw	TCAGAACGCGTAGTTAAAGTTAGTACCGAACAGCCAGGCTTTACCTTCAG*TAATACGACTCACTATAGGGCTC*
ompA-Up	ATGAAAAAGACAGCTATCGCGATTGCAGTGGCACTGGCTGGTTTCGCTAC*AATTAACCCTCACTAAAGGGCG*
ompA-Dw	TTAAGCCTGCGGCTGAGTTACAACGTCTTTGATACCTTTAACTTCGATCT*TAATACGACTCACTATAGGGCTC*
ompC-Up	ATGAAAGTTAAAGTACTGTCCCTCCTGGTCCCAGCTCTGCTGGTAGCAGG*AATTAACCCTCACTAAAGGGCG*
ompC-Dw	TTAGAACTGGTAAACCAGACCCAGAGCTACGATGTTATCAGTGTTGATGC*TAATACGACTCACTATAGGGCTC*
fhuA-Up	ATGGCGCGTTCCAAAACTGCTCAGCCAAAACACTCACTGCGTAAAATCGC*AATTAACCCTCACTAAAGGGCG*
fhuA-Dw	TTAGAAACGGAAGGTTGCGGTTGCAACGACCTGACGTTCTGCGCCCCAGA*TAATACGACTCACTATAGGGCTC*
fepA-Up	ATGAACAAGAAGATTCATTCCCTGGCCTTGTTGGTCAATCTGGGGATTTA*AATTAACCCTCACTAAAGGGCG*
fepA-Dw	TCAGAAGTGGGTGTTTACGCTCATATACCACGTACGTCCCGGCTCGTTAT*TAATACGACTCACTATAGGGCTC*
btuB-Up	CCGCTTGGGCACAGGATACCAGCCCGGATACTCTCGTCGTTACTGCTAACC*AATTAACCCTCACTAAAGGGCG*
btuB-Dw	ATCGATCAAGTCACTGACATCGTTACGATATCCGGAAATACGCCAGTTCA*TAATACGACTCACTATAGGGCTC*
tolC-Up	ATGAAGAAATTGCTCCCCATTCTTATCGGCCTGAGCCTTTCTGGGTTCAG*AATTAACCCTCACTAAAGGGCG*
tolC-Dw	TCAGTTACGGAAAGGGTTATGACCGTTACTGGTGGTAGTGCGTGCGGATG*TAATACGACTCACTATAGGGCTC*

lamB-Up	ATGATGATTACTCTGCGCAAACTTCCTCTGGCGGTTGCCGTCGC AGCGGG*AATTAACCCTCACTAAAGGGCG*
lamB-Dw	TTACCACCAGATTTCCATCTGGGCACCGAAGGTCCACTCGTCGC TGTCGC*TAATACGACTCACTATAGGGCTC*

Table S4: List of primers for control of the deletion of genes expressing common bacteriophage receptor genes in *E. coli* strain ECOR4, related to Figure 5.

**Primers for deletion control**	
ompW-F	ATGAAAAAGTTAACAGTGGCG
ompW-R	TTAAAAACGATATCCTGCTGAGAG
tsx_F	ATGAAAAAAACATTACTGGCAGCC
tsx_R	TCAGAAGTTGTAACCTACTACCAGG
waaC_F	ATGCGGGTTTTGATCGTTAAAACATC
waaC_R	TTAAATCAATGTGATTTTTTTATAAACAGCCG
tonB-F	ATGACCCTTGATTTACCTCGC
tonB-R	TTACTGAATTTCGGTGGTGC
waaR_F	ATGAATGAATTTATAAAAGAACGGTTTTCG
waaR_R	TTATTTCTTAAGCTTGTACTTAATTAATGAAGTTA
ompF_F	ATGATGAAGCGCAATATTCTGGC
ompF_R	TTAGAACTGGTAAACGATACCCACAG
fadL_F	ATGAGCCAGAAAACCCTGTTTACAAAG
fadL_R	TCAGAACGCGTAGTTAAAGTTAGTAC
ompA_F	ATGAAAAAGACAGCTATCGCGATTG
ompA_R	TTAAGCCTGCGGCTGAGTTACAAC
ompC_F	ATGAAAGTTAAAGTACTGTCCCTCC
ompC_R	TTAGAACTGGTAAACCAGACCCAG
fhuA_F	ATGGCGCGTTCCAAAACTGC
fhuA_R	TTAGAAACGGAAGGTTGCGGTTG
fepA-F	ATGAACAAGAAGATTCATTCCCTG
fepA-R	TCAGAAGTGGGTGTTTACGC
btuB_F	ATGATTAAAAAAGCTTCGCTGCTGAC
btuB_R	TCAGAAGGTGTAGCTGCCAGACAAG
tolC_F	ATGAAGAAATTGCTCCCCATTCTTATC
tolC_R	TCAGTTACGGAAAGGGTTATGACC

lamB_F	ATGATGATTACTCTGCGCAAACTTC
lamB_R	TTACCACCAGATTTCCATCTGGG

Figure S1: Protein sequence alignment of gp38 corresponding to the chaperone encoded by T4, related to Figure 5.
Differing residues are highlighted in red colour.

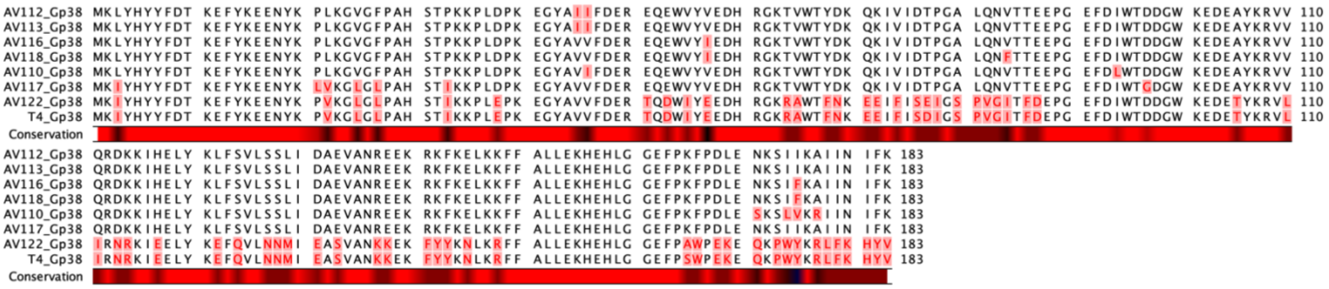

Figure S2 Protein sequence alignment of the tail fibers corresponding to the adhesins encoded by gp38 homologues of the phages T2 and T6, related to Figure 5. Differing residues are highlighted in red colour.

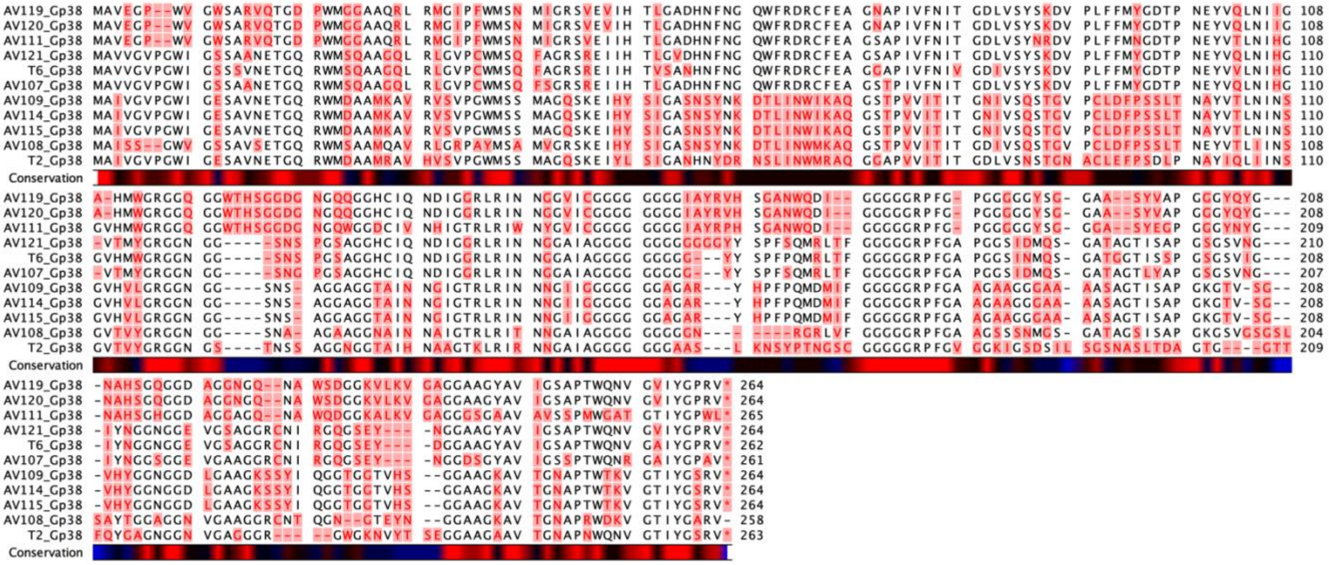

Figure S3. Protein sequence alignment of gp37 of T4 and full sequences of gp37 of phage T4 and AV110, AV112, AV113, AV116, AV117, AV118, AV122, related to Figure 5. Differing residues are highlighted in red.

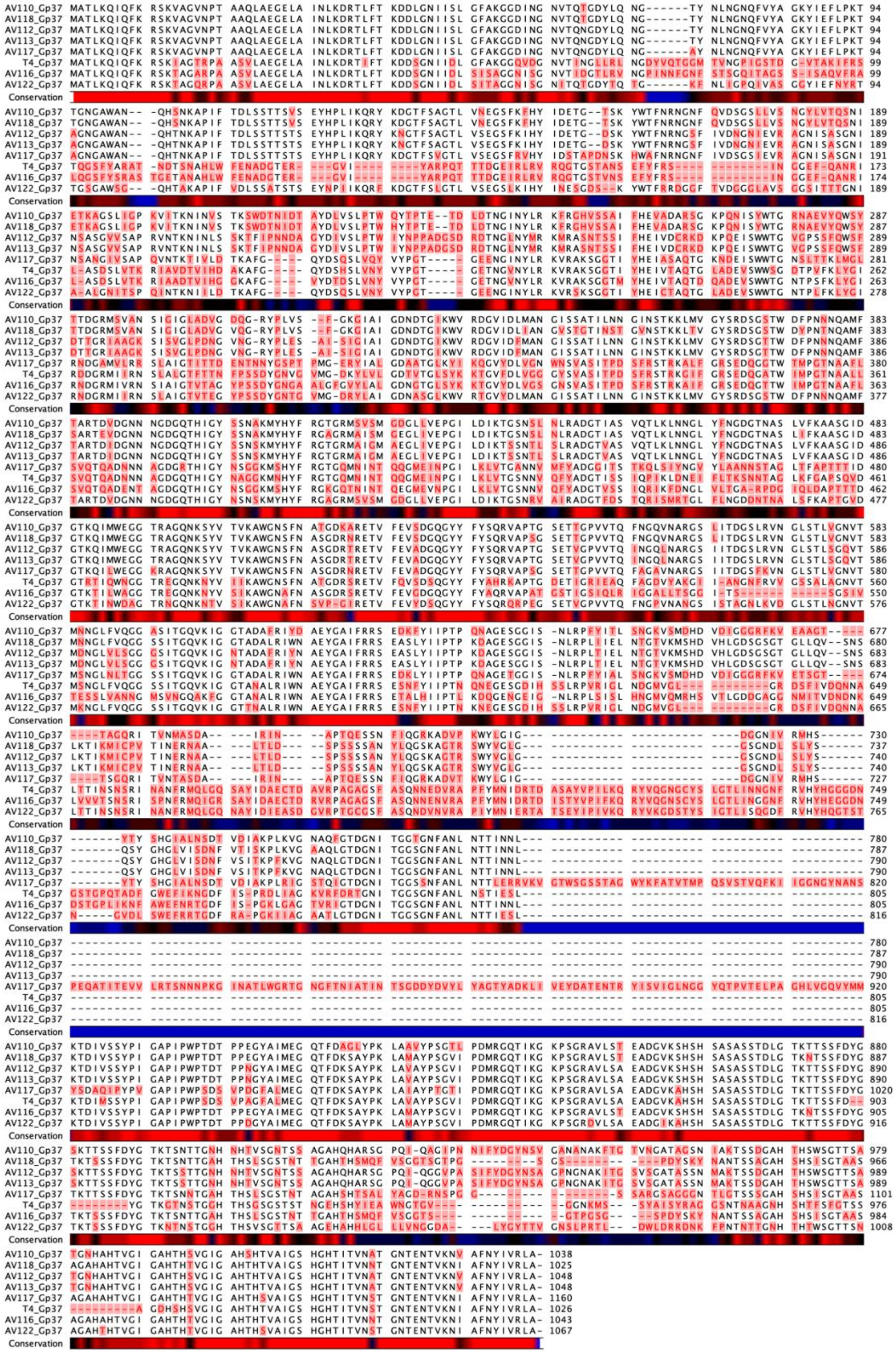

Supplement: Document S1. Figures S1–S3 and Tables S2–S4 [file mmc1.pdf]
